# Sea Ice Dynamics Drive Benthic Microbial Communities in McMurdo Sound, Antarctica

**DOI:** 10.3389/fmicb.2021.745915

**Published:** 2021-10-28

**Authors:** Ashleigh A. Currie, Alexis J. Marshall, Andrew M. Lohrer, Vonda J. Cummings, Sarah Seabrook, S. Craig Cary

**Affiliations:** ^1^School of Science, University of Waikato, Hamilton, New Zealand; ^2^Environmental Research Institute, International Centre for Terrestrial Antarctic Research, Hamilton, New Zealand; ^3^National Institute of Water and Atmosphere, Hamilton, New Zealand; ^4^National Institute of Water and Atmosphere, Wellington, New Zealand

**Keywords:** sediment, benthic, microbial, community, climate change, sea ice, organic matter

## Abstract

Climate change is driving dramatic variability in sea ice dynamics, a key driver in polar marine ecosystems. Projected changes in Antarctica suggest that regional warming will force dramatic shifts in sea ice thickness and persistence, altering sea ice-associated primary production and deposition to the seafloor. To improve our understanding of the impacts of sea ice change on benthic ecosystems, we directly compared the benthic microbial communities underlying first-year sea ice (FYI) and multi-year sea ice (MYI). Using two tractable coastal habitats in McMurdo Sound, Antarctica, where FYI (Cape Evans) and MYI (New Harbour) prevail, we show that the structure and composition of the benthic microbial communities reflect the legacy of sea ice dynamics. At Cape Evans, an enrichment of known heterotrophic algal polysaccharide degrading taxa (e.g., *Flavobacteriaceae*, unclassified *Gammaproteobacteria*, and *Rubritaleaceae*) and sulfate-reducing bacteria (e.g., *Desulfocapsaceae*) correlated with comparatively higher chlorophyll *a* (14.2±0.8μgg^−1^) and total organic carbon content (0.33%±0.04), reflecting increased productivity and seafloor deposition beneath FYI. Conversely, at New Harbour, an enrichment of known archaeal (e.g., *Nitrosopumilaceae*) and bacterial (e.g., *Woeseiaceae* and *Nitrospiraceae*) chemoautotrophs was common in sediments with considerably lower chlorophyll *a* (1.0±0.24μgg^−1^) and total organic carbon content (0.17%±0.01), reflecting restricted productivity beneath MYI. We also report evidence of a submarine discharge of sub-permafrost brine from Taylor Valley into New Harbour. By comparing our two study sites, we show that under current climate-warming scenarios, changes to sea ice productivity and seafloor deposition are likely to initiate major shifts in benthic microbial communities, with heterotrophic organic matter degradation processes becoming increasingly important. This study provides the first assessment of how legacy sea ice conditions influence benthic microbial communities in Antarctica, contributing insight into sea ice–benthic coupling and ecosystem functioning in a polar environment.

## Introduction

High-latitude polar oceans are covered in an extensive layer of sea ice, which grows and shrinks throughout seasonal cycles. Covering 3–6% of Earth’s surface area (Comiso, 2003), the high albedo of sea ice and snow cover reflects large fractions of incoming solar radiation, playing a crucial role in regulating Earth’s global climate system (Hall, 2004). However, the anthropogenic increase in atmospheric greenhouse gas concentrations is increasing Earth’s surface temperature and driving variability in sea ice dynamics (e.g., Thomas and Dieckmann, 2008). In the Arctic, the once dominant thicker and more persistent multi-year sea ice (MYI) is declining and transitioning into thinner and more ephemeral first-year sea ice (FYI; Polyakov et al., 2012; Meier et al., 2014). Recent climate models have estimated that within the Arctic, an ice-free summer is likely to occur before the end of this century (Overland and Wang, 2013; Notz and Stroeve, 2018). In the Antarctic, an overall gradual increase in sea ice extent was observed from 1979 to 2014, with the greatest increases occurring in the Ross Sea. However, since 2014, strong declines in overall Antarctic sea ice extent have greatly exceeded rates in the Arctic, with a record low recorded in 2017 (Maksym, 2019; Parkinson, 2019). Current climate models predict that substantial continental scale losses of sea ice will occur in the Antarctic before the end of this century (Bracegirdle et al., 2008; Collins et al., 2013; Smith et al., 2014).

Sea ice provides an important habitat for diverse under-ice microalgal communities that are typically dominated by diatoms (Arrigo, 2014). Primary productivity is closely associated with variations in sea ice conditions and its control on sunlight availability, consequently influencing the amount of under-ice algal biomass and detrital material falling to the seafloor (Dayton et al., 1986; Lizotte, 2001; Boetius et al., 2013). Once settled on the seafloor, the algal-derived organic matter serves as a major food resource to benthic primary consumers (Norkko et al., 2007; Wing et al., 2012, 2018). Predictions for the Antarctic suggest that declines in sea ice and snow cover will lead to increased primary productivity (Lohrer et al., 2013; Smith et al., 2014; Leung et al., 2015; Kaufman et al., 2017). There have been observations of rapid and long-lasting reorganizations of benthic macrofauna (Thrush and Cummings, 2011; Dayton et al., 2013; Clark et al., 2017) associated with changes in sea ice conditions and organic matter deposition. However, much less is known about the effects on benthic microbial communities, the key drivers of organic matter remineralization processes and biogeochemical cycling (Falkowski et al., 2008). Previous polar studies have shown that the quality and quantity of organic matter are major drivers of microbial diversity and community composition, with certain taxonomic groups showing strong affiliations with environmental parameters such as sediment algal pigment concentration and total organic carbon (TOC; Bienhold et al., 2012; Ruff et al., 2014; Learman et al., 2016; Cho et al., 2020). However, few studies in Antarctica have characterized the benthic microbial communities, and none have compared the effects of FYI and MYI, which may provide a unique perspective on marine ecosystem functioning in these difficult to study and rapidly changing polar environments.

The Ross Sea represents the southernmost seasonally ice-free ocean region in the world, and sea ice dynamics in this region have so far been among the slowest on Earth to react to climate change – unlike regions further north, such as the Antarctic Peninsula (Kim et al., 2018). The McMurdo Sound, in the southwestern Ross Sea (77°S latitude), is covered by land-fast sea ice (ice fastened to the coastline) for most of the year. Sea ice thickness and persistence conditions within McMurdo Sound differ across the east and west sides which, along with circulation patterns, drive regions of low and high primary productivity, generating measurable differences in the supply of labile organic matter to the seafloor and benthic macrofauna assemblages (Dayton and Oliver, 1977; Barry, 1988; Thrush et al., 2006). In this study, we used the strong east–west food supply gradients at Cape Evans and New Harbour as tractable means of determining whether the structure and diversity of the benthic microbial communities are reflective of FYI and MYI conditions, respectively. Due to the proximity of New Harbour to the terminus of the Taylor Valley, one of the McMurdo Dry Valleys, we also considered the potential influence of local terrestrial inputs on the microbial communities at New Harbour. Terrestrial surface material is known to be transported by katabatic winds down the valley to the surface of the near-shore MYI (Murray et al., 2013). Additionally, we investigated the hypothesis proposed by Mikucki et al. (2015) that a sub-permafrost brine aquifer underlying the Taylor Valley discharges at New Harbour by investigating the presence of extreme halophiles.

## Materials and Methods

### Study Sites, Sampling, and Sediment Analysis

**Figure 1:**
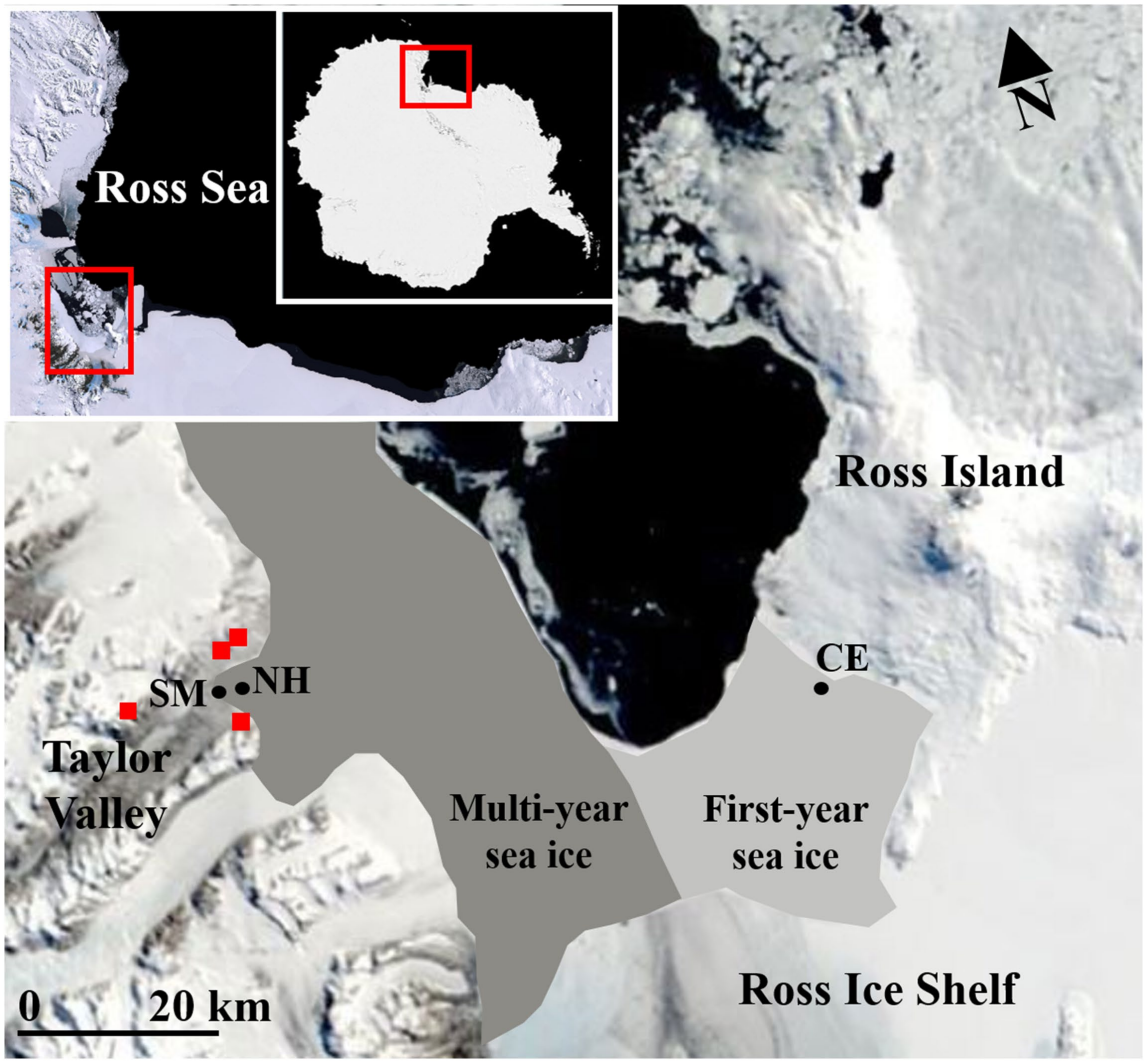
Moderate Resolution Imaging Spectroradiometer (MODIS) satellite imagery of McMurdo Sound and McMurdo Ice Shelf region during November 2017 with sample site locations [Cape Evans, CE (14.2m); Explorers Cove, New Harbour, NH (18.5m); the shoreline moat, SM (~0.1m)]. The locations for four New Zealand Terrestrial Antarctic Biocomplexity Survey (nzTABS) terrestrial soil samples within the lower Taylor Valley are represented by red squares. Regions historically dominated by multi-year sea ice (dark grey) vs. first-year sea ice (light grey) are overlaid on the map (indicative only). Brackish water is present at the shoreline moat site during months of sunlight. Image credit: MODIS, NASA, United States.

The microbial communities inhabiting the surface sediments of the seafloor were sampled from Explorers Cove, New Harbour (NH; 77°34.573ʹS, 163°32.608ʹE), and Cape Evans (CE; 77°38.115ʹS, 166°24.410ʹE) in western and eastern McMurdo Sound, respectively (Figure 1). Two sites were sampled by divers (NH, 18.5m depth; CE, 14.2m depth) who accessed the seafloor through holes in the sea ice (3.5 and 2.0m thick at NH and CE, respectively). Additionally, sediments were collected from the edge of a shoreline moat (SM, ~0.1m depth) at the beach-moat interface of Explorers Cove, at the eastern terminus of Taylor Valley in the McMurdo Dry Valleys (Figure 1). The water of the shoreline moat is brackish and forms during spring and summer as temperatures increase. All samples were collected by the same personnel during the same expedition (November 2017) using 2-cm internal diameter by 2-cm deep cores (*n*=4 or 5 randomly positioned replicates from a single 20-m transect per site). A total of 13 cores were collected: four each from Cape Evans and the shoreline moat and five from Explorers Cove, New Harbour. After collection, each sediment sample was homogenized using a sterile spatula, and a 5-ml subsample was transferred into a new 15-ml Falcon tube. Falcon tubes were centrifuged for 5min at 1,000 RCF, and the supernatant discarded. At least 5ml of LifeGuard Soil DNA Preservation Solution (Qiagen, CA, United States) was added to each tube to preserve DNA. The tubes were capped, shaken by hand to mix, and stored at −20°C until nucleic acid extraction.

Sediment samples from Cape Evans and New Harbour were prepared for analyses of sediment pigments (chlorophyll *a* and its degradation product pheophytin), TOC, total nitrogen (TN), and δ^13^C and δ^15^N stable isotopic composition following previously published protocols (Kennedy et al., 2005). Pigments were extracted from freeze-dried sediments in 90% ethanol, and their concentrations measured spectrophotometrically. For the remainder, 5g of wet sediment per sample was dried in a 40°C oven in an aluminum pan, and then homogenized by grinding with a mortar and pestle. Carbonate was removed using repeated treatments of 1M HCl until samples stopped effervescing, with drying occurring in a 40°C oven between each addition. An Isoprime 100 analyzer (Elementar, Langenselbold, Germany) was used for TOC and TN measurements and a 20/20 isotope analyzer (Sercon Ltd., Crewe, United Kingdom) for isotopic abundance at the Waikato Stable Isotope Unit (WSIU; Hamilton, New Zealand).

### Amplicon Sequencing for Microbial Community Analysis

A modified DNA extraction protocol was developed using the Qiagen PowerSoil®DNA Isolation Kit (CA, United States). Briefly, for each sample, five tubes each containing 200mg of wet sediment were extracted and then pooled together onto a single elution column to concentrate the extracted material. The protocol was modified at the “prepare sample” step (C1 buffer heated to 60°C), “cell lysis” step (samples heated on a Thermomixer for 10min at 60°C then shaken on a vortex mixer for 10s before bead beating), “inhibitor removal technology” step (incubation time increased to 10min), “bind DNA” step (incubation time increased to 20min, samples inverted every 3min), “wash” step (five representative extracts from each sample were pooled through the same spin column), and “elute” step (DNA eluted in 20μl of 60°C nuclease-free water and left on the spin column at room temperature for 2min before centrifuging). DNA concentration was determined using a Qubit 2.0 Fluorometer (Life Technologies, CA, United States) and the quality confirmed by electrophoresis in 2% TAE agarose gel prior to downstream analysis.

The 16S hypervariable region V4–V5 was PCR amplified in triplicate reactions using the primer set 515F-Y and 926R (Quince et al., 2011; Parada et al., 2016) modified for Ion Torrent sequencing using a one-step PCR strategy (IonCode Barcode Adapters, Thermo Fisher Scientific). For each sample, amplicons were generated in triplicate in 20-μl reaction volumes of 4.8mM dNTP (Invitrogen, CA, United States), 1×PCR buffer, 120mM MgCl_2_ (Invitrogen), 4mM forward and reverse primer (Integrated DNA Technologies, Inc., IA, United States), 1U Taq DNA polymerase (Invitrogen), and 3ng total genomic DNA with the following conditions: 3-min initial denaturation at 94°C, followed by 30cycles of 94°C for 45s, 50°C for 1min, and 72°C for 1.5min, and a final elongation step at 72°C for 10min. Triplicate reactions were pooled and PCR amplicon products normalized using a SequalPrep Normalization Plate Kit (Invitrogen). Amplicons were sequenced using Ion Torrent Personal Genome Machine (PGM) DNA sequencer chemistry with an Ion 318v2 chip (Life Technologies) at the University of Waikato DNA sequencing facility (Hamilton, New Zealand).

We were fortunate to be able to use raw sequencing data from Taylor Valley soil samples, used under permission from the New Zealand Terrestrial Antarctic Biocomplexity Survey (nzTABS).^1^ DNA was extracted from Taylor Valley soil samples using a previously published CTAB method (Power et al., 2018). The V4 region of the 16S rRNA was amplified in triplicate using 515F-806R primers (Caporaso et al., 2011) and sequenced as described above.

### Raw Sequence Data Processing

To understand the impact of changing sea ice conditions on benthic microbial communities, we had to ensure potential terrestrial inputs from the Taylor Valley were not influencing the microbial communities at New Harbour. To do this, we constructed two independent datasets. We constructed Dataset 1 to identify terrestrial impacts on the marine sediment microbiomes and Dataset 2 to assess the influence of sea ice thickness and persistence on the benthic microbial communities at Cape Evans and New Harbour.

Sequencing adapters, low-quality reads and short reads (< 250 bp) were identified and removed with Mothur v1.40.5 (Schloss et al., 2009). Ion Torrent barcodes and sequencing primers were removed, and new labels were created using the Python script fastq_strip_barcode_relabel2.py in USEARCH v10 (Edgar, 2010).

A total of 539,109 valid reads across nine samples (Dataset 1) and a total of 127,668 valid reads across nine samples (Dataset 2) were processed using DADA2 v1.14.1 (Callahan et al., 2016) in R v3.6.3 (R Core Team, 2018) to generate amplicon sequence variants (ASVs; Supplementary Table 1). Briefly, reads < 250 bp, quality score < 2, and expected error > 2 were removed. After all quality steps, an ASV table was constructed containing 4,649 ASVs and 3,037 ASVs with an average read length of 225 bp for Dataset 1 and 2, respectively. Representative ASV sequences were used to check for chimeras, and 312 and 56 chimeric sequences were removed for Dataset 1 and 2, respectively. A total of 4,394 and 2,981 unique ASVs were produced for analysis for Dataset 1 and 2, respectively. Taxonomy was assigned to ASVs using the SILVA v138 database (Quast et al., 2012). Sequences were aligned using Multiple Alignment using Fast Fourier Transform (MAFFT) v7 (Katoh et al., 2002), and a phylogenetic tree was generated using FastTree v2.1.11 (Price et al., 2010).

ASVs classified as eukaryotes, mitochondria, chloroplasts, or those sequences that were unclassified at the level of kingdom and phylum were removed from the datasets. Before discarding chloroplast ASVs, these sequences were classified using Basic Local Alignment Search Tool (BLAST) against the nt database. Raw sequence data can be accessed at the Sequence Read Archive under the BioProject accession ID PRJNA721518.

### Sequencing Data Analyses

Analysis of ASVs was completed in R v3.6.3 with data visualized using ggplot2 v3.3.0 (Wickham, 2016).^2^

#### Dataset 1 Sequencing Data Analyses

The rarefaction curves were calculated to confirm completeness of sequencing using the R wrapper ggrare^3^ and displayed using ggplot2. Breakaway v4.6.10 (Willis and Bunge, 2015) was used to estimate total species richness (observed plus unobserved) and Shannon diversity (Shannon, 1948) within the bacterial and archaeal communities at all four sample sites. Species richness was calculated at the level of ASVs. All ASVs classified to the level of domain were kept within the dataset, and species richness was estimated on all ASVs classified to this level. The statistical differences in taxonomic diversity values between sample sites were assessed for each metric using Wilcoxon rank-sum tests.

A principal coordinates analysis (PCoA) ordination of weighted and unweighted UniFrac dissimilarity matrices based on transformed data was used in Phyloseq v1.30.0 (McMurdie and Holmes, 2013) to determine the community compositional dissimilarity (beta diversity) between all four sample sites. Analysis of similarity (ANOSIM; Clarke, 1993) was determined using vegan v2.5 (Oksanen et al., 2020) to test the significance of the differences identified by PCoA between sampling units.

#### Dataset 2 Sequencing Data Analyses

The rarefaction curves were calculated to confirm completeness of sequencing using the R wrapper ggrare and displayed using ggplot2. Breakaway v4.6.10 was used to estimate total species richness (observed plus unobserved) and Shannon diversity (Shannon, 1948) within the bacterial and archaeal communities at Cape Evans and New Harbour. Species richness was calculated at the level of ASVs. All ASVs classified to the level of domain were kept within the dataset, and species richness was estimated on all ASVs classified to this level. The statistical differences in taxonomic diversity values between sample sites were assessed for each metric using Wilcoxon rank-sum tests.

Phylogenetic clustering and overdispersion were measured for Cape Evans and New Harbour samples in Picante v1.8.1 (Kembel et al., 2010) using divergence-based measures of mean pairwise distance (MPD; relatedness of species deep in the tree; Webb, 2000) and mean nearest taxon distance (MNTD; relatedness near branch tips; Webb et al., 2002). MPD and MNTD were standardized to account for differences in species richness between sites, resulting in a nearest relative index (NRI) and nearest taxon index (NTI), respectively (Webb, 2000). A positive NRI/NTI value indicates phylogenetic clustering in which coexisting taxa are more closely related than expected by chance. A negative NRI/NTI value indicates phylogenetic overdispersion in which coexisting taxa are more distantly related than expected by chance.

A PCoA ordination of weighted and unweighted UniFrac dissimilarity matrices based on transformed data was used in Phyloseq v1.30.0 to determine the community compositional dissimilarity (beta diversity) between Cape Evans and New Harbour sample sites. ANOSIM (Clarke, 1993) was determined using vegan v2.5 (Oksanen et al., 2020) to test the significance of the differences identified by PCoA between sampling units.

To identify ASVs with differing relative abundances between Cape Evans and New Harbour, the analysis of composition of microbiomes (ANCOM) v1.1-3 (Mandal et al., 2015) was applied with default settings. ASVs identified as significant (*p*<0.05) by ANCOM were grouped together at the level of family. Those family groupings with cumulative relative abundances <1.5% were removed from the dataset to focus the interpretation and heat map visualization on more abundant and site-specific family groupings.

Functional gene abundances were predicted for ASVs in Cape Evans and New Harbour samples using PICRUSt2 v2.3.0 beta (Phylogenetic Investigation of Communities by Reconstruction of Unobserved States; Douglas et al., 2020; using tools: EPA-NG (Barbera et al., 2019) and Gappa (Czech et al., 2020) for phylogenetic placement of reads, Castor (Louca and Doebeli, 2018) for hidden state prediction, and MinPath (Ye and Doak, 2009) for pathway inference). The nearest sequenced taxon index (NSTI) was used to estimate accuracy of PICRUSt2 predictions, and ASVs with a NSTI value >2.0 were removed. Averaged abundances of predicted pathways for each site derived by PICRUSt2 from the Metabolic Pathway Database (MetaCyc; Caspi et al., 2020) were selected, and a two-sided z-test statistically compared the predicted MetaCyc functions between sample sites in STAMP v2.1.3 (STatistical Analysis of Metagenomic Profiles; Parks et al., 2014). The Newcombe-Wilson method was used to calculate the confidence interval (95%) and features with <2 reads, and *p*>0.05 was removed. We selected MetaCyc pathways of interest based on expected dominant biogeochemical pathways in coastal marine sediment environments subjected to variations in productivity [sulfate reduction I (assimilatory), nitrate reduction VI (assimilatory), nitrate reduction I (denitrification), nitrifier denitrification, reductive tricarboxylic acid (rTCA) cycle I, reductive TCA cycle II, Calvin–Benson–Bassham (CBB) cycle, and glycine betaine degradation I] to compare function across sites.

## Results

### Sediment Characteristics

The concentration of sediment chlorophyll *a* (a proxy for fresh algal material) and pheophytin (a proxy for degraded algal material) at Cape Evans was 14× higher than that of New Harbour (Table 1). The ratio of fresh-to-degraded algal material (chlorophyll *a*/pheophytin) was 2:1 at both study sites. The δ^13^C and δ^15^N values were similar between Cape Evans and New Harbour sediments. Sediment TOC from Cape Evans was higher than New Harbour, as was TN. TOC/TN ratios of the sediments were 7.1±0.3 at Cape Evans and 8.5±0.5 at New Harbour.

**Table 1:** Sea ice and surface sediment characteristics at Cape Evans (17/11/2017) and New Harbour (7/11/2017).

Sample site	Cape Evans	New Harbour
Date sampled (dd/mm/yyyy)	17/11/2017	7/11/2017
Sea water temperature (°C)	−0.85 max	−1.69 max
	−1.90min	−1.96min
**Sea ice characteristics**		
Thickness (m)	2.0	3.5
Snow cover	Minimal	Minimal
Surface	Smooth	Smooth
Persistence	First-year	Multi-year
Last fast-ice breakout	Feb–Mar (2017)	Feb–Mar (2016)
**Sediment geochemistry**		
Chlorophyll *a* content (μgg^−1^ sediment)	14.2±0.8	1.0±0.24
Pheophytin content (μgg^−1^ sediment)	7.2±0.25	0.5±0.14
% Total organic carbon (TOC)	0.33±0.04	0.17±0.01
% Total nitrogen (TN)	0.05±0.01	0.02±0.00
δ^13^C (‰)	−20.26±0.01	−21.90±0.73
δ^15^N (‰)	4.15±0.23	4.31±0.68

*All sediment geochemistry data given as mean±1 SE (n=4 and 5, respectively)*.

### Identifying Terrestrial Impacts on the Marine Sediment Microbiomes

Due to the proximity of the New Harbour sampling site to the Taylor Valley, it was important that we demonstrate that wind-blown terrestrial inputs were not influencing the marine sediment microbiomes so we could be confident that any differences between Cape Evans and New Harbour microbial communities would be in relation to the impact of local FYI and MYI conditions.

Sequencing sampling effort was assessed for all sequences recovered from Cape Evans, New Harbour, shoreline moat and the Taylor Valley (Supplementary Figure 1A). Sample read counts ranged from 7,794 to 77,707 per sample (Supplementary Table 1). Total ASVs classified as eukaryotes (120 ASVs), mitochondria (38 ASVs), chloroplasts (41 ASVs), or that were unclassified at the level of kingdom (21 ASVs) and phylum (190 ASVs) were removed from the dataset. The filtered and quality checked 16S rRNA gene amplicon dataset included a total of 3,851 ASVs across all 17 samples from Cape Evans, New Harbour, the shoreline moat, and Taylor Valley.

Taxonomic classification of ASVs across the samples from Cape Evans, New Harbour, the shoreline moat, and Taylor Valley identified that at the level of phylum, *Actinobacteriota* (52.8%±4.9) and *Bacteroidota* (11.7%±2.5) dominated Taylor Valley (Supplementary Figure 2A), whereas Cape Evans, New Harbour, and the shoreline moat were dominated by *Proteobacteria* (CE 33.5%±1.8; NH 39.7%±1.5; SM 24.5%±2.3) and *Bacteroidota* (CE 29.8%±1.4; NH 14.8%±2.3; SM 36.8%±4.1). *Planctomycetota* (CE 10.0%±0.7; NH 14.7%±1.7) were also common at Cape Evans and New Harbour. The shoreline moat was the only site also dominated by *Cyanobacteria* (12.4%±2.1).

At the level of class, Taylor Valley was dominated by *Thermoleophilia* (23.8%±6.4), *Actinobacteria* (13.7%±2.8), and *Bacteroidia* (11.6%±2.6; Supplementary Figure 2B). Cape Evans, New Harbour, and the shoreline moat were all dominated by *Bacteroidia* (CE 29.5%±1.6; NH 14.1%±2.4; SM 36.3%±4.2) and *Gammaproteobacteria* (CE 25.7%±1.4; NH 25.8%±1.6; SM 12.6%±1.6). *Alphaproteobacteria* (NH 13.7%±1.2; SM 11.9%±1.0) were also common at New Harbour and the shoreline moat. New Harbour was the only site where *Planctomycetes* (13.5%±1.6) dominated, and the shoreline moat was the only site where *Cyanobacteria* (12.4%±2.1) dominated.

Significant site-specific compositional differences were identified across all four sampling sites. The most significant compositional differences occurred between the Taylor Valley soils and the marine sediments collected from the other three sites, with 43.5% of the variation explained across PCoA axis 1 (ANOSIM R=0.9, *p*<0.01, weighted UniFrac; Supplementary Figure 2C). The structure of the microbial communities collected from the Taylor Valley and the shoreline moat was distinct from the communities collected from New Harbour and Cape Evans (Supplementary Figure 2C). A separation between Cape Evans and New Harbour samples occurred across PCoA axis 2 which explained 14.4% of the variation (Supplementary Figure 2C).

Estimates of species richness were similar across Cape Evans, New Harbour, and the shoreline moat sites (CE 1,942.20±156.85; NH 2,041.21±117.74; SM 1,845.21±267.88), whereas Taylor Valley was comparably lower (1,179.35±326.02; Supplementary Figure 2D). Shannon diversity was the highest in New Harbour samples (*Hʹ* 6.22±0.02) compared to all other sites (CE *Hʹ were similar across* 5.78±0.20; SM *Hʹ* 6.06±0.13) and Taylor Valley was the lowest (*Hʹ* 4.77±0.65; Supplementary Figure 2E).

### Influence of Sea Ice Thickness and Persistence on the Benthic Microbial Communities at Cape Evans and New Harbour

Sequencing sampling effort were assessed for all sequences recovered from Cape Evans and New Harbour (Supplementary Figure 1B). Sample read counts ranged from 7,779 to 20,001 per sample (Supplementary Table 1). Total ASVs classified as eukaryotes (115 ASVs), mitochondria (31 ASVs), chloroplasts (39 ASVs), or that were unclassified at the level of kingdom (18 ASVs) and phylum (144 ASVs) were removed from the datasets. Chloroplast ASVs represented 5,198 reads in the dataset (2%), and BLAST analysis identified these reads to be predominantly diatoms *Haslea nipkowii* (43.5%), *Skeletonema pseudocostatum* (22.7%), *Chaetoceros* sp. (9.0%), and *Asterionellopsis glacialis* (5.1%).

The filtered and quality checked 16S rRNA gene amplicon datasets included a total of 2,076 ASVs across nine samples from Cape Evans and New Harbour. Of these ASVs, 1,308 ASVs (63%) were found at both Cape Evans and New Harbour.

Taxonomic classification of ASVs within the sediment samples across Cape Evans and New Harbour identified that at the phylum level, *Proteobacteria* (CE 34.0%±1.9; NH 40.5%±1.6), *Bacteroidota* (CE 30.1%±1.5; NH 15.3%±2.4), and *Planctomycetota* (CE 10.2%±0.7; NH 15.1%±1.7) dominated the communities. *Verrucomicrobiota* (CE 13.2%±1.8; NH 7.5%±1.2) were also common at both sites.

**Figure 2:**
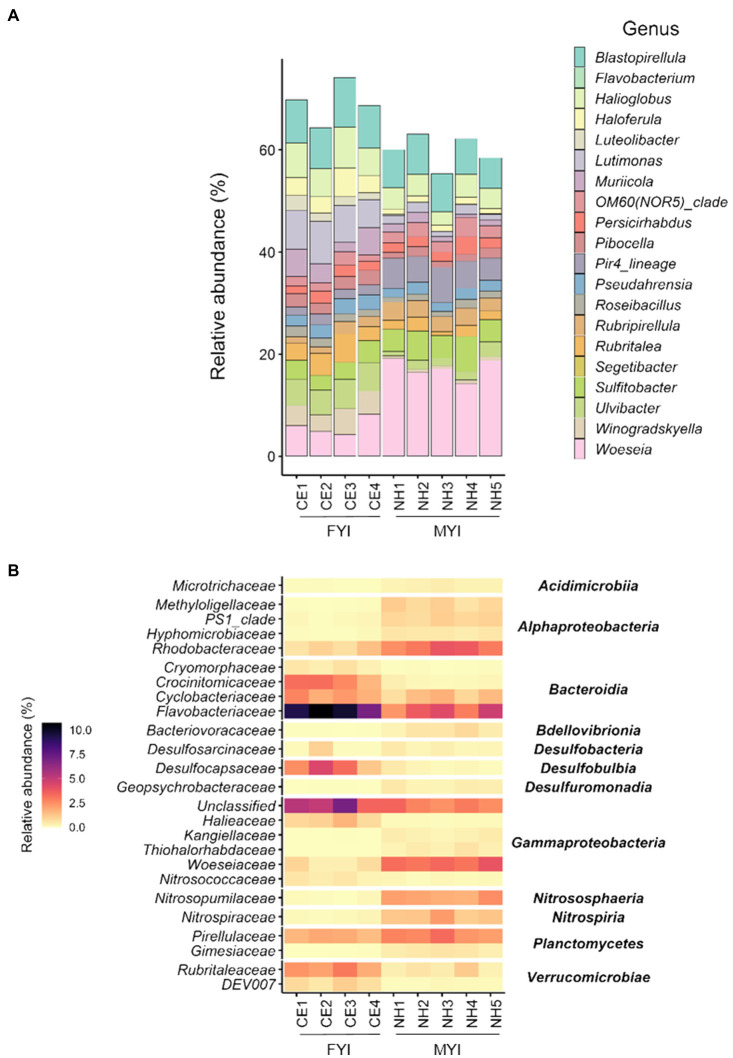
Microbial community composition at the level of genus **(A)** in surface sediment samples from Cape Evans (CE) and New Harbour (NH). Differentially abundant taxa identified by ANCOM (analysis of composition of microbiomes; **(B)** in surface sediment samples from Cape Evans and New Harbour, with *p*>0.05 and relative sequence abundance >1.5%, visualized in a heatmap at the taxonomic level of family (left column) and order (right column).

At the level of genus, *Woeseia* (CE 5.8%±1.8; NH 17.1%±2.0) and *Blastopirellula* (CE 8.6%±0.7; NH 7.1%±0.8) were both dominant at Cape Evans and New Harbour (Figure 2A). Additionally, *Lutimonas* (7.2%±1.2), *Halioglobus* (6.4%±1.2), and *Ulvibacter* (5.3%±0.4) were dominant at Cape Evans, and Pir4_lineage (5.5%±1.0) and *Sulfitobacter* (5.1%±1.1) were dominant at New Harbour.

Our search for extreme halophiles identified the presence of *Thiohalorhabdales* (CE 0.5%±0.2; NH 1.5%±0.6) and *Geopsychrobacteraceae* (CE 0.0%±0.0; NH 0.4%±0.1) at Cape Evans and New Harbour. *Thiohalorhabdales* were not present in the samples exposed to air (shoreline moat or Taylor Valley).

**Figure 3:**
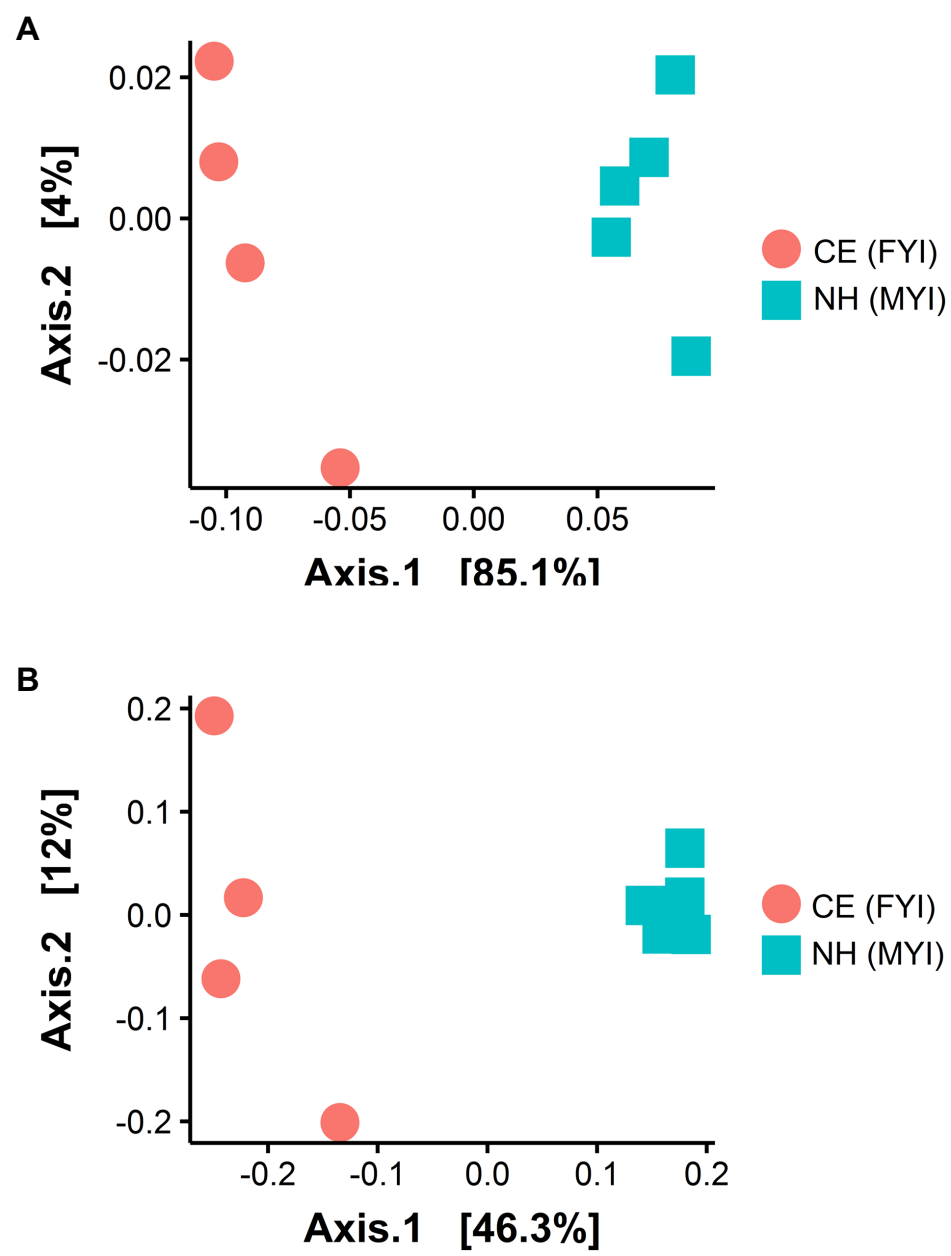
Beta diversity in surface sediment samples from Cape Evans (CE, red circle) and New Harbour (NH, blue square) using weighted UniFrac (analysis of similarity, ANOSIM=0.9, *p*<0.01; **(A)** and unweighted UniFrac (ANOSIM=0.9, *p*<0.01; **(B)** dissimilarity matrices.

Significant site-specific compositional differences were identified across Cape Evans and New Harbour sampling sites with 85.1% of the variation explained across PCoA axis 1 (ANOSIM *R*=0.9, *p*<0.01, weighted UniFrac; Figure 3A) and 46.3% of the variation explained across PCoA axis 1 (ANOSIM R=0.9, *p*<0.01, unweighted UniFrac; Figure 3B).

**Figure 4:**
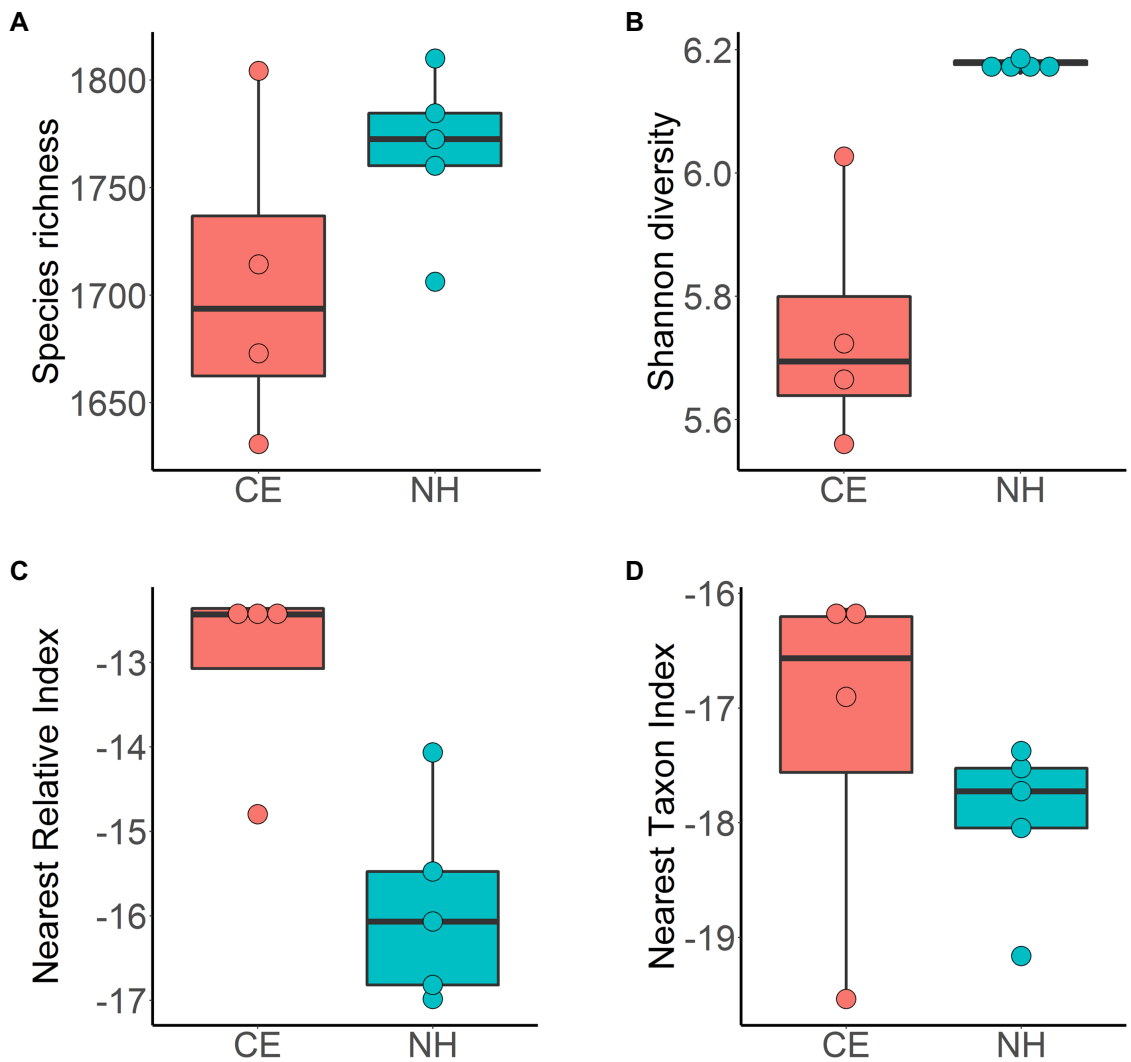
Alpha diversity of microbial communities in surface sediment samples from Cape Evans (CE, red) and New Harbour (NH, blue) estimated using metrics of species richness **(A)**, Shannon–Wiener index **(B)**, the nearest relative index **(C)**, and the nearest taxon index **(D)**. Horizontal bar represents value of sample median, box represents the interquartile range, and whiskers represent maximum and minimum values.

Estimates of species richness were not significantly different between sites (CE 1,705.62±74.08; NH 1,766.71±38.47, *p*=NS; Figure 4A). Shannon diversity was higher in New Harbour samples (*Hʹ* 6.18±0.01) than in Cape Evans samples (*Hʹ* 5.74±0.20; *p*<0.05; Figure 4B). The phylogenetic diversity measured by the NRI and NTI revealed negative values across both sites with more negative values in New Harbour samples (−15.83±0.75 and −19.11±0.87, respectively) than in Cape Evans samples (−13.56±0.42 and −17.49±1.05, respectively; Figures 4C,D).

The relative sequence abundances of 218 ASVs differed between Cape Evans and New Harbour (ANCOM *p*<0.05; Supplementary Table 2). These 218 ASVs were grouped at the level of family and were reflected by 51 distinct families. Across these ASVs, those families with accumulative relative abundances of >1.5% were displayed (Figure 2B), reflective of 25 families. Of the 97 ASVs identified at Cape Evans, the most differentially abundant family level taxa were represented by *Flavobacteriaceae* (13 ASVs; 9.2%), unclassified groups of *Gammaproteobacteria* (nine ASVs; 5.3%), *Desulfocapsaceae* (five ASVs; 3.0%), *Crocinitomicaceae* (two ASVs, 2.6%), and *Rubritaleaceae* (seven ASVs; 2.4%; Figure 2B). Of these differentially abundant ASVs, the genera *Lutimonas* (two ASVs, 3.5%), *Ulvibacter* (one ASV, 1.7%), and *Winogradskyella* (two ASVs, 1.7%) dominated the *Flavobacteriaceae*; *Haloferula* (two ASVs, 1.2%) and *Luteolibacter* (three ASVs, 0.7%) dominated the *Rubritaleaceae*; and unclassifiable genera (two ASVs, 1.6%) and SEEP-SRB4 (one ASV, 0.8%) dominated the *Desulfocapsaceae*. *Crocinitomicaceae* were unclassifiable at the level of genus.

Of the 122 significant ASVs identified as more abundant at New Harbour, the dominant family level taxa were represented by *Woeseiaceae* (11 ASVs; 3.4%), *Flavobacteriaceae* (five ASVs; 3.2%), *Rhodobacteraceae* (nine ASVs; 3.2%), *Pirellulaceae* (14 ASVs; 2.5%), *Nitrosopumilaceae* (four ASVs; 2.0%), and *Nitrospiraceae* (four ASVs; 1.4%; Figure 2B). At the level of genus, the differentially abundant *Woeseiaceae* ASVs were dominated by *Woeseia* (11 ASVs, 3.4%), *Pirellulaceae* were dominated by Pir4 lineage (five ASVs, 1.1%) and *Blastopirellula* (five ASVs, 0.7%), *Rhodobacteraceae* were dominated by NAC11-7 lineage (two ASVs, 1.0%) and *Roseobacter* (one ASV, 0.8%), and *Nitrospiraceae* were dominated by *Nitrospira* (four ASVs, 1.4%; Supplementary Table 2). The differentially abundant *Flavobacteriaceae* and *Nitrosopumilaceae* ASVs at New Harbour were unclassified at the level of genus.

In association with the hypothesized submarine discharge of sub-permafrost brine from the Taylor Valley, the extreme halophile *Thiohalorhabdales* was also identified as being a differentially abundant family level taxa at New Harbour (two ASVs; 0.4%) compared to Cape Evans. The *Thiohalorhabdales* ASVs were unclassified at the level of genus.

Using PICRUSt2 to assign metabolic function from taxonomic inference, a total of 245 MetaCyc pathways were inferred within the Cape Evans and New Harbour samples following the removal of 29 ASVs which had NSTI scores of >2. Of the pathways of interest selected across the two study sites, PICRUSt2 predicted that the differences in relative pathway abundances for sulfate reduction I (assimilatory) and the reductive TCA cycle II were higher at Cape Evans than New Harbour (Supplementary Figure 3), whereas at New Harbour, predictions of the relative pathway abundances for the reductive TCA cycle I, CBB cycle, nitrate reduction I (denitrification), nitrifier denitrification, and glycine betaine degradation I pathways were higher than at Cape Evans (Supplementary Figure 3).

## Discussion

McMurdo Sound in the southwestern Ross Sea exhibits contrasting sea ice conditions that drive regions of low vs. high primary productivity (Dayton and Oliver, 1977; Lohrer et al., 2013), providing a unique setting to investigate and compare the impact of sea ice thickness and persistence on shallow-water coastal benthic microbial communities and their potential role as sentinels for climate change. Land-fast FYI breaks out annually in the east at Cape Evans, and the area is typically ice-free from mid-December to late March, whereas thicker MYI in the west at New Harbour rarely breaks out. Sea ice dynamics at these two sites are well-known to influence food supply, benthic faunal densities, community composition and food web structure (Dayton and Oliver, 1977; Kim et al., 2019), and microbially mediated rates and processes (Lohrer et al., 2013). Here, we show that there is a strong association between the legacy of sea ice dynamics and benthic microbial community structure and composition. With Antarctic and Arctic sea ice predicted to decline this century (Bracegirdle et al., 2008; Collins et al., 2013; Overland and Wang, 2013; Smith et al., 2014; Notz and Stroeve, 2018), this highlights the capacity of benthic microbial communities to be sentinels for climate change within polar marine ecosystems.

We found that the origin and quantity of organic matter on the seafloor at Cape Evans and New Harbour reflected the legacy of local sea ice dynamics. The measured stable isotopes of carbon within the sediments at both sites (δ^13^C –20.3‰ to −22.7‰) were suggestive that the origin of the organic matter was likely to be the same at both sites [i.e., diatoms (−20.3‰; Meyers, 1994)], and were comparable to sea ice associated δ^13^C microbial communities previously measured at Cape Evans [−20.2‰ (Norkko et al., 2007); −19.8‰ (Wing et al., 2018)]. Sediment δ^13^C at Cape Evans and New Harbour was marginally lower than that previously reported (−18.7‰ and −19.7‰, respectively) and higher than δ^13^C found in pelagic phytoplankton (−23.8‰ and −24.0‰, respectively; Norkko et al., 2007). When combined, these results indicate that settling sea ice algae provided the primary source of organic matter to the benthos at both sites at the time of sampling, not advected phytoplankton from the water column. This finding supports previous observations that organic matter originating from sea ice is the primary food source for benthic macrofauna under sea ice at Cape Evans (Dayton et al., 1986; Norkko et al., 2007; Wing et al., 2018). We cannot rule out that some primary production does come from benthic diatoms. However, Wing et al. (2018) and Norkko et al. (2007) have also previously reported that sea ice-associated algae is the primary source of organic matter to the benthos within McMurdo Sound.

The impact of FYI vs. MYI on the sediment environment was evident in the concentrations of chlorophyll *a*, pheophytin, TOC, and TN, which were higher at the FYI site (Cape Evans) than the MYI site (New Harbour). These differences reflect the legacy of ice thickness and persistence at these two sites as sea ice conditions (including ice opacity and snow cover) affect sea ice algal productivity, the flux of ice algal-derived organic matter to the seafloor, and *in situ* microphytobenthos production (Dayton et al., 1986; Norkko et al., 2007; Thrush and Cummings, 2011). Consistent with higher chlorophyll *a* concentration and TOC content, heterotrophic bacteria associated with phytoplankton blooms and which participate in the degradation of algal polysaccharides (Teeling et al., 2012; Buchan et al., 2014; Cardman et al., 2014; Krüger et al., 2019) were enriched at Cape Evans compared to New Harbour. For example, Cape Evans sediments had higher abundances of *Flavobacteriaceae*, which are well-known specialists in the initial break down of algal-derived organic matter (Teeling et al., 2012; Buchan et al., 2014; Krüger et al., 2019). Studies in the Antarctic Polar Front (Ruff et al., 2014), the Antarctic Peninsula (Learman et al., 2016; Li et al., 2020), and the Mertz Glacier Polynya, East Antarctica (Bowman and McCuaig, 2003) support the role of *Flavobacteriaceae*, including the genus *Ulvibacter*, in the degradation of diatom-derived organic matter. The sloughing of ice algae from the under-ice layer and melting of the FYI itself at Cape Evans could also play a role in structuring the benthic microbial community, with the relative sequence abundance of taxa associated with sinking algal aggregates [e.g., *Ulvibacter* and *Winogradskyella* (Rapp et al., 2018)] higher than in New Harbour. Taxa within the family *Rubritaleaceae* (genera *Haloferula* and *Luteolibacter*) were also more common at Cape Evans. An Arctic fjord (Smeerenburgfjord, Svalbard) study suggested that both *Haloferula* and *Luteolibacter* were associated with the breakdown of laminarin, the major storage glucan in diatoms (Cardman et al., 2014). Laminarin is one of the most well-studied algal polysaccharides and contributes substantially to carbon export in the ocean (Becker et al., 2020). Together, these taxa may also be playing a central role within the community at Cape Evans by breaking down complex algal polysaccharides.

In support of increased organic matter deposition at Cape Evans, strictly anaerobic sulfate-reducing bacteria [e.g., *Desulfocapsaceae* (CE 4.0%±1.5; NH 0.6%±0.5)] were more abundant at this site compared to New Harbour and the PICRUSt2 pathway prediction tool identified that this community had a greater capacity for sulfate reduction I (assimilatory). Higher organic matter deposition rates and increased degradation of organic matter can result in greater compression of redox zones which affects microbial community distribution (Rasigraf et al., 2020). It is therefore likely that the anoxic sediment layer is shallower at Cape Evans, and that anaerobic organic matter degradation processes are more important within this community than at New Harbour. Altogether, our results suggest that at Cape Evans, the legacy of annual FYI breakout and intense pulse fluxes of organic matter have selected for heterotrophic taxa that specialize in degrading algal detrital material within the sediments. In regions where MYI is predicted to breakout more frequently, heterotrophic benthic bacteria are likely to play an increasingly important role in transferring energy through the food web.

At New Harbour, windblown terrestrial sediment is known to accumulate on the near-shore MYI at the terminus of the ice-free Taylor Valley (Murray et al., 2013), thus it was important that we assess the possible influence of terrestrial inputs on the benthic microbial community at New Harbour. Our analysis showed that the composition of the microbial community collected from sediment within the shoreline moat, where the MYI meets the Taylor Valley coastline, was composed of taxa that reflected both terrestrial (Taylor Valley) and marine (New Harbour) input. For example, similarities with the Taylor Valley included the presence of *Actinobacteria*, *Blastocatellia*, and *Thermoleophilia*, whereas the presence of *Gammaproteobacteria* and *Planctomycetes* reflect more marine influences as would be expected in an intertidal marine environment impacted by katabatic Taylor Valley winds. These observations show that aeolian inputs have had minimal impact on the composition of the microbial community at our New Harbour marine site, consistent with the findings by Chewings et al. (2014) who calculated low sedimentation rates of aeolian material in New Harbour. Nevertheless, accumulations of terrestrial sediments within and on the surface of the MYI in Explorers Cove, while very evident, is also very patchy (authors’ pers. obs.) and more widespread seafloor sampling is recommended to confirm the consistency of this observation.

In comparison with Cape Evans, the presence of MYI and low organic inputs at New Harbour selected for a community enriched in chemolithoautotrophic taxa. New Harbour had comparatively higher abundances of taxa capable of ammonia oxidation [e.g., *Nitrosopumilaceae* (Könneke et al., 2014)] and nitrite oxidation [e.g., *Nitrospira* (Daims et al., 2015)], and those with known metabolic potential for sulfur and hydrogen oxidation [e.g., *Woeseiaceae* (Mußmann et al., 2017; Hoffmann et al., 2020)]. Our PICRUSt2 analysis also predicted higher representations of CO_2_ fixation pathways at New Harbour including the rTCA cycle I and CBB cycle, and included predictions for a higher representation of the nitrate reduction VI (assimilatory) and nitrifier denitrification pathways. The enrichment of ammonia-oxidizing *Nitrosopumilaceae* in the low organic matter sediments at New Harbour was consistent with reports that this archaeal group thrives in nutrient limited environments (Könneke et al., 2014) including deep-sea sediments of the Ross Sea (Learman et al., 2016). Deep-sea sediment studies in the Guaymas Basin, Gulf of California (Baker et al., 2013) and North Pond, Mid-Atlantic Ridge (Zhao et al., 2019) also suggested the role of nitrite-oxidizing bacteria *Nitrospira* as important contributors to nitrogen cycling, carrying out the second step of nitrification coupled to CO_2_ fixation *via* the rTCA cycle (Lücker et al., 2010; Bayer et al., 2020). The higher relative importance of chemoautotrophy at New Harbour was further supported by the enrichment of *Woeseiaceae* (previously JTB225 Marine Benthic Group) which are prevalent in deep-sea sediments (Bienhold et al., 2016; Dyksma et al., 2016) and are capable of CO_2_ fixation *via* the CBB cycle (Mußmann et al., 2017; Hoffmann et al., 2020). Consistent with lower sediment chlorophyll *a* and TOC at New Harbour, it is likely that these chemolithoautotrophic taxa have adapted to life under a legacy of limited organic resource availability by specializing in using alternative inorganic energy sources. Thus, the selection of these taxa at New Harbour is reflective of the legacy of the prevailing MYI conditions. Under current climate-change scenarios, the role that chemolithoautotrophic taxa play in supporting primary productivity within the community may become less important if organic matter fluxes increase.

In 2015, Mikucki et al. (2015) proposed that a sub-permafrost brine system may exist in the Taylor Valley, discharging at New Harbour. In searching for known extreme halophiles within the dataset, we identified two halophiles [*Thiohalorhabdales* (NH 1.5%±0.6; CE 0.5%±0.2) and *Geopsychrobacteraceae* (NH 0.4%±0.1; CE 0.0%±0.0)] that were significantly more abundant at New Harbour than at Cape Evans, lending taxonomic support for the presence of sub-surface brine. Given that we showed the communities at New Harbour were significantly more diverse than at Cape Evans, these differences between relative sequence abundances of *Thiohalorhabdales* and *Geopsychrobacteraceae* across sites are noteworthy. PICRUSt2 also predicted increased glycine betaine degradation I pathways at New Harbour compared to Cape Evans, a compound widely used by the majority of bacteria for osmoprotection (Sleator and Hill, 2002). Extreme halophiles grow in high-osmolarity environments, and their presence at New Harbour supports the proposal of a submarine discharge of sub-permafrost brines from the Taylor Valley. This finding is also consistent with earlier work of the Dry Valley Drilling Project in the 1970s, during which boreholes drilled 183m below sea level near the shoreline of New Harbour filled with liquid brine (Cartwright and Harris, 1981). Further investigations of the subsurface hydrology of the McMurdo Dry Valleys and the associated microbial communities are needed to better understand the degree and implications of such hypothesized connectivity between the two systems.

Our analyses show that the legacy of local sea ice dynamics has a strong influence on the diversity of the benthic microbial communities at Cape Evans and New Harbour. Our observation of increased diversity at New Harbour compared to Cape Evans is potentially reflective of a more stable environment driven by a history of MYI cover and low productivity vs. a more dynamic environment driven by ephemeral and productive FYI. Both the NRI and NTI also revealed greater phylogenetic overdispersion (coexisting taxa are more distantly related than expected by chance) within the communities at New Harbour than at Cape Evans. The low temperatures, prolonged darkness, and consistently low nutrient inputs at New Harbour are analogous to conditions of the deep-sea (Dayton and Oliver, 1977). In deep-sea sediments, infaunal communities exhibit remarkably high diversity despite only intermittent fluxes of detrital material falling from the water column (Hessler and Sanders, 1967). More recently, microbial communities were also shown to be extremely diverse in deep-sea sediments, such as the eastern South Atlantic Ocean (Schauer et al., 2010), the Pacific Ocean in the Arctic (Li et al., 2009), and Fram Strait in the Arctic (Jacob et al., 2013). It has been theorized that the co-existence of many species in deep-sea sediments arises from past competitive interactions within a stable environment, thus promoting niche differentiation and enabling many taxa with narrower metabolic niches to co-exist (Sanders, 1968). Our observations support those by Dayton and Oliver (1977), and we propose that the persistent MYI conditions and low carbon inputs at New Harbour, which provide stable conditions that parallel the deep-sea, selects for more diverse and specialized microbial taxa. These findings support our overarching hypothesis that the legacy of sea ice conditions structure the benthic microbial communities at Cape Evans and New Harbour.

Future climate change scenarios predict substantial sea ice decline this century in both the Antarctic and Arctic (Bracegirdle et al., 2008; Collins et al., 2013; Overland and Wang, 2013; Smith et al., 2014; Notz and Stroeve, 2018; IPCC, 2019). Based on the taxonomic assessment made in this study, increased fluxes of organic matter to the seafloor may cause significant community shifts with heterotrophic taxa (e.g., *Flavobacteriaceae*, unclassified *Gammaproteobacteria*, and *Rubritaleaceae*) becoming more dominant and playing an increasingly important role in transferring energy through the food web. An enrichment of *Flavobacteriaceae* at New Harbour may be a sign that change is already underway, as sea ice breakout events at this site have been increasing in frequency recently (authors’ pers. obs.). Changes to microbial community structure and functioning are likely to affect nutrient cycling between the sediments and water column, with unknown effects on primary production and marine ecosystem functioning. We highlight the role of the connectivity between sea ice and shallow water benthic microbial communities and demonstrate the importance of microorganisms as sentinels of climate change (Cavicchioli et al., 2019) in polar marine ecosystems globally.

## Data Availability Statement

The datasets presented in this study can be found in online repositories. The names of the repository/repositories and accession number(s) can be found at: https://www.ncbi.nlm.nih.gov/genbank/, PRJNA721518.

## Author Contributions

The project was conceived and designed by SC, AL, VC, and AM. Support for the project was obtained by SC, AL, and VC. Samples were collected by AL, and all subsequent laboratory work and analyses were conducted by AC and AM. The manuscript was written by AC, AM, and SC with contribution and editing by SS, AL, and VC. All authors contributed to the article and approved the submitted version.

## Funding

This research was conducted as part of the Resilience in Antarctic Biota and Ecosystems Project, funded by the New Zealand Antarctic Research Institute (RFP2016-2) to SC, VC, and AL. Support for field logistics was provided by Antarctica New Zealand. Support for AC came directly through a University of Waikato Postgraduate Scholarship. AM was supported by Smart Ideas Award (UOWX1602) from the New Zealand Ministry of Business, Innovation and Employment and the Rutherford Foundation Royal Society Te Aparangi Postdoctoral Fellowship (20-UOW006).

## Conflict of Interest

The authors declare that the research was conducted in the absence of any commercial or financial relationships that could be construed as a potential conflict of interest.

## Publisher’s Note

All claims expressed in this article are solely those of the authors and do not necessarily represent those of their affiliated organizations, or those of the publisher, the editors and the reviewers. Any product that may be evaluated in this article, or claim that may be made by its manufacturer, is not guaranteed or endorsed by the publisher.

## Footnotes

^1^ https://ictar.aq/nztabs-science/

^2^ https://github.com/ThermophileResearchUnit/Currie_etal_submission.git

^3^ https://rdrr.io/github/gauravsk/ranacapa/man/ggrare
