# Supplementary material for: Sea Ice Dynamics Drive Benthic Microbial Communities in McMurdo Sound, Antarctica
Source: Front Microbiol. 2021 Oct 28;12:745915. doi: 10.3389/fmicb.2021.745915 (PMC8581541; doi:10.3389/fmicb.2021.745915)
Supplement: Supplementary file 1 [file Data_Sheet_1.PDF]

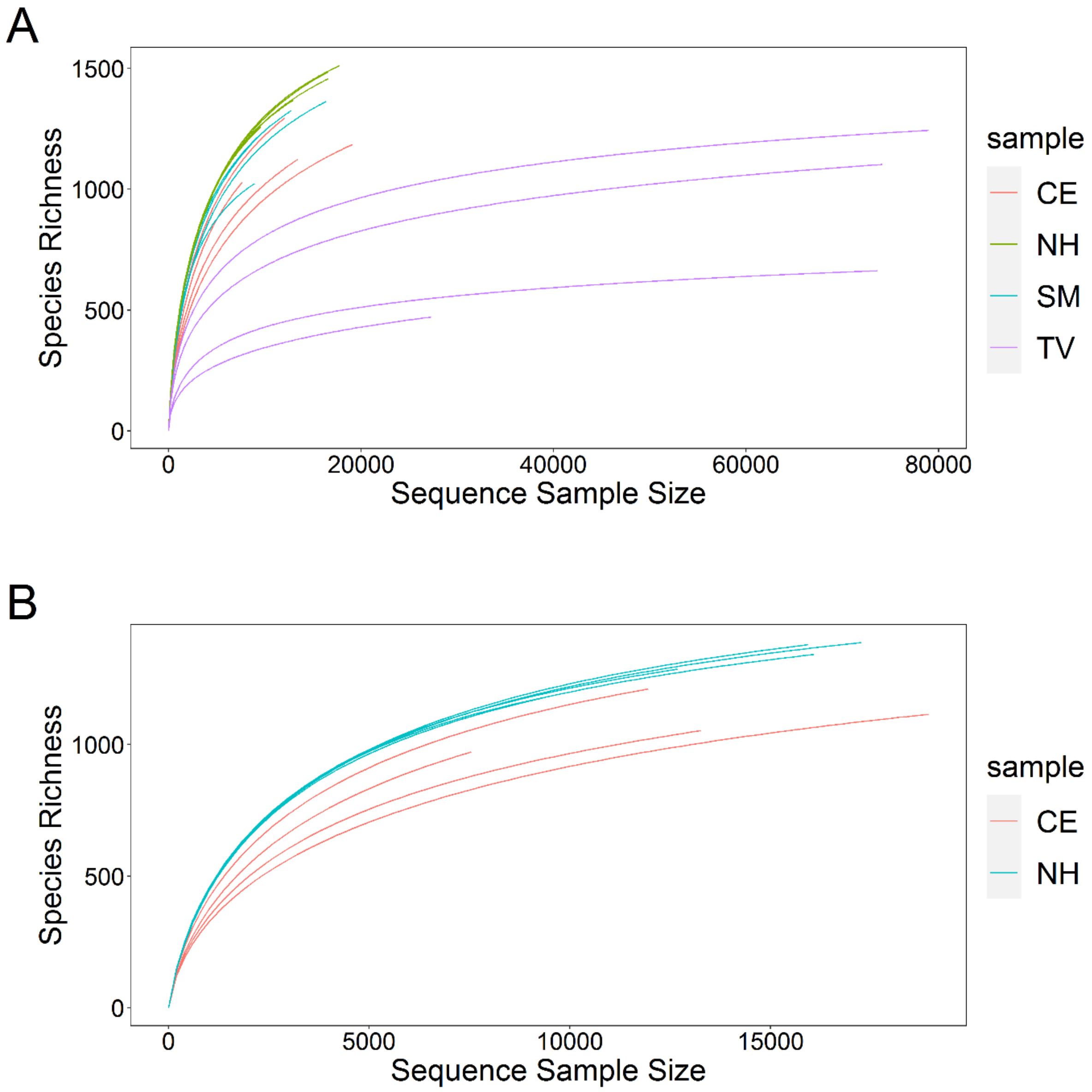

Supplementary Figure 1. Rarefaction curves based on 16S rRNA sequencing of the microbial communities indicating the observed number of ASVs at (A) Cape Evans (CE), shoreline moat (SM), New Harbour (NH), and Taylor Valley (TV), and (B) only Cape Evans (CE) and New Harbour (NH).

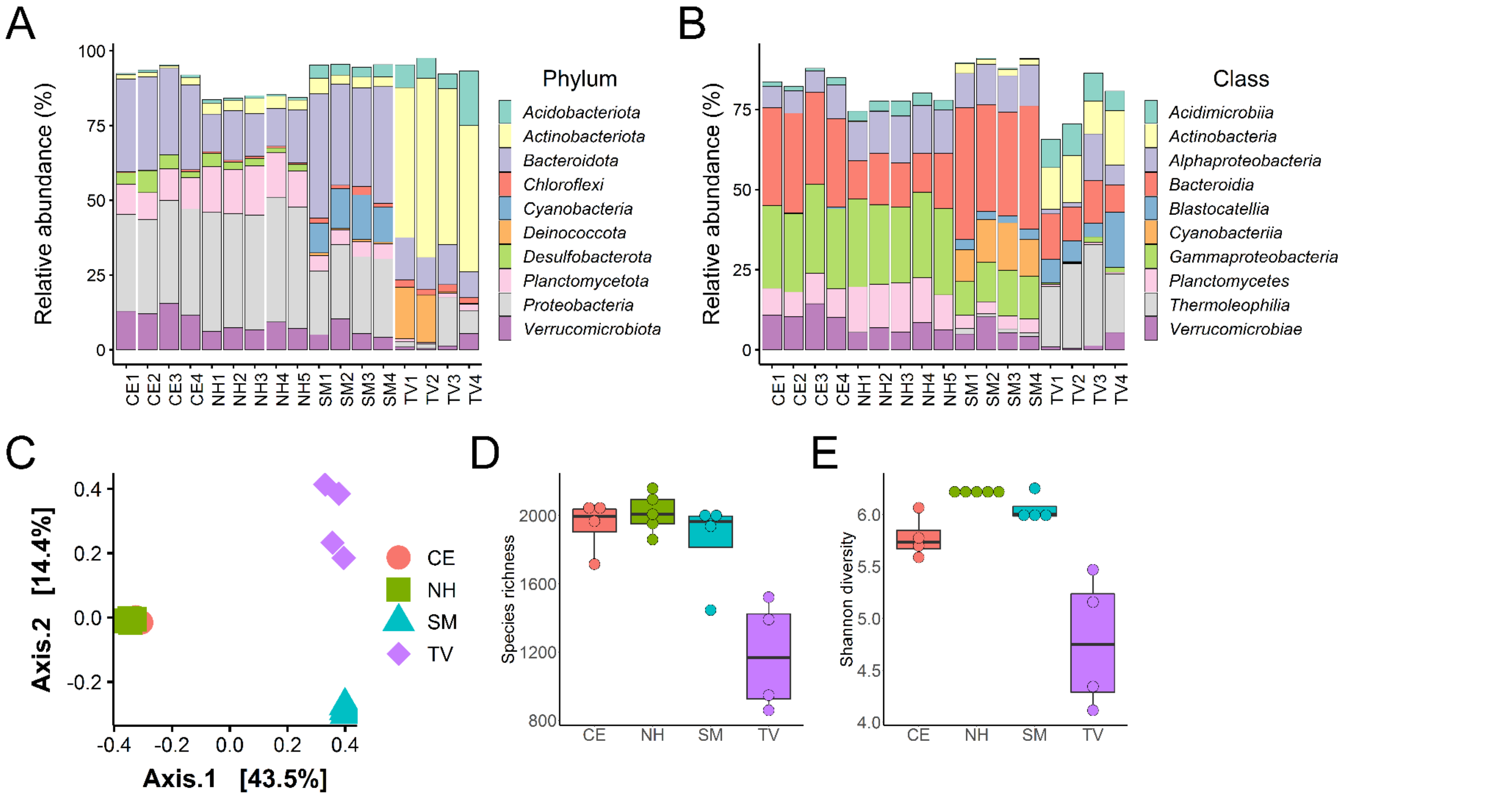

Supplementary Figure 2. Microbial community composition at the levels of phylum (A) and class (B), and unweighted UniFrac beta diversity (C; ANOSIM = 0.9, $p < 0.01$) in surface sediment samples from Cape Evans, New Harbour, the shoreline moat, and the terrestrial Taylor Valley. Alpha diversity estimated using metrics of species richness (D) and Shannon-Wiener index (E). Horizontal bar represents value of sample median, box represents the interquartile range, and whiskers represent maximum and minimum values. CE = Cape Evans; SM = shoreline moat; NH = New Harbour; TV = Taylor Valley.

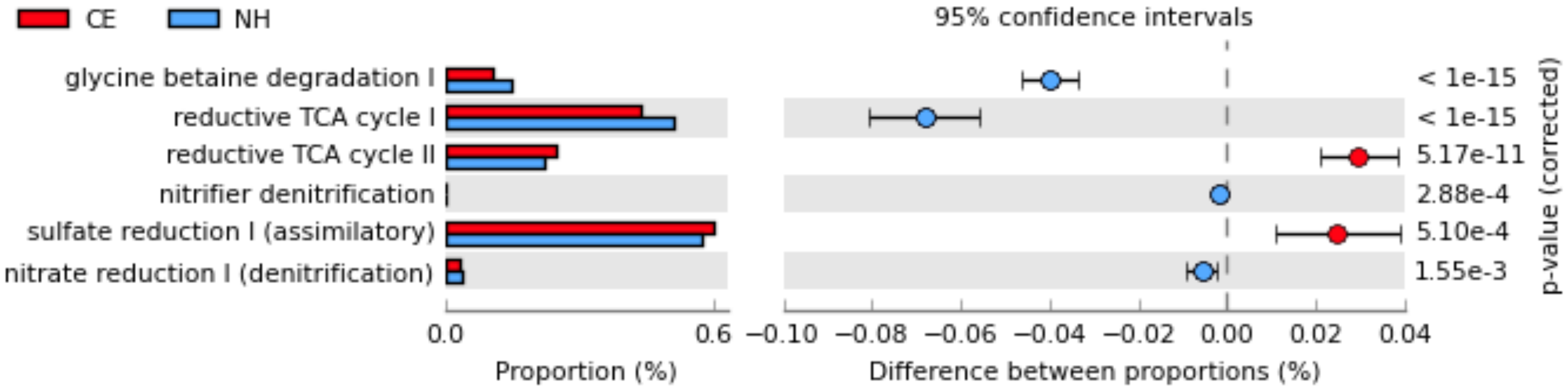

Supplementary Figure 3. Functionally predicted MetaCyc pathways of interest at Cape Evans and New Harbour shown in a bar plot as proportions of differential pathways predicted by PICRUSt2. The difference between proportions between the groups is shown with 95% confidence intervals. Only p value <0.05 are shown.

Supplementary Table 1. Raw sequence read counts, and sequence read counts post quality filtering

Dataset	Sample ID	Initial sequences	Sequences after filtering and trimming	Sequences after denoising	Sequences after chimera removal (read counts)	Number of ASVs
1	Cape Evans 1	11266	9053	8542	7794	1041
	Cape Evans 2	19546	15593	14852	13639	1142
	Cape Evans 3	27323	22187	21294	19255	1193
	Cape Evans 4	17002	13915	13071	12185	1303
	New Harbour 1	13336	10689	10194	9958	1273
	New Harbour 2	22752	18413	17611	17131	1501
	New Harbour 3	17322	13958	13333	13114	1381
	Hew Harbour 4	22881	18548	17514	17077	1475
	New Harbour 5	24984	20301	19375	18903	1525
	Shoreline Moat 1	11131	9451	9197	9112	1043
	Shoreline Moat 2	21572	17607	16727	16603	1391
	Shoreline Moat 3	16749	13758	12984	12874	1347
	Shoreline Moat 4	13763	11350	10825	10700	1042
	Taylor Valley 1	87117	77657	75284	73395	668
	Taylor Valley 2	30772	28794	28216	27299	473
	Taylor Valley 3	87988	77212	76395	74432	1109
	Taylor Valley 4	93605	84036	81185	77707	1259
2	Cape Evans 1	11266	9053	7865	7779	1047
	Cape Evans 2	19546	15593	14182	14065	1139
	Cape Evans 3	27323	22187	20587	20001	1188
	Cape Evans 4	17002	13915	12355	12221	1289
	New Harbour 1	13336	10689	9422	9328	1255
	New Harbour 2	22752	18413	16658	16621	1480
	New Harbour 3	17322	13958	12529	12521	1374
	Hew Harbour 4	22881	18548	16670	16666	1458
	New Harbour 5	24984	20301	18522	18466	1503

Supplementary Table 2. Differentially abundant ASVs identified by ANCOM (Analysis of Composition of Microbiomes) in surface sediment samples from Cape Evans (CE) and New Harbour (NH), with *p* <0.05 and relative sequence abundance >1%.

ASV	Family	Genus	Relative sequence abundance (%)	
			CE	NH
ASV_1007	*Anaerolineaceae*	Unclassified	0.00	0.06
ASV_746	*Anaerolineaceae*	Unclassified	0.00	0.08
ASV_747	*Anaerolineaceae*	Unclassified	0.08	0.00
ASV_972	*Arenicellaceae*	Unclassified	0.00	0.06
ASV_141	*Bacteriovoracaceae*	Unclassified	0.01	0.51
ASV_362	*Bacteroidetes_BD2-2*	Unclassified	0.11	0.00
ASV_667	*Blastocatellaceae*	*Blastocatella*	0.07	0.00
ASV_812	*Caldilineaceae*	Unclassified	0.02	0.00
ASV_164	*Chromatiaceae*	*Halochromatium*	0.26	0.00
ASV_13	*Crocinitomicaceae*	Unclassified	1.82	0.15
ASV_53	*Crocinitomicaceae*	Unclassified	0.82	0.00
ASV_117	*Cryomorphaceae*	*Cryomorpha*	0.43	0.04
ASV_792	*Cryomorphaceae*	*Vicingus*	0.05	0.00
ASV_1338	*Cyclobacteriaceae*	Unclassified	0.05	0.00
ASV_14	*Cyclobacteriaceae*	Unclassified	1.78	0.12
ASV_1996	*Cyclobacteriaceae*	Unclassified	0.03	0.00
ASV_43	*Cyclobacteriaceae*	Unclassified	0.04	1.17
ASV_443	*Cyclobacteriaceae*	Unclassified	0.15	0.00
ASV_452	*Cyclobacteriaceae*	Unclassified	0.07	0.00
ASV_796	*Defluviicoccaceae*	*Defluviicoccus*	0.08	0.00
ASV_549	*Desulfobacteraceae*	Unclassified	0.12	0.00
ASV_269	*Desulfocapsaceae*	*Desulfopila*	0.15	0.00
ASV_5	*Desulfocapsaceae*	Unclassified	1.12	0.06
ASV_62	*Desulfocapsaceae*	*SEEP-SRB4*	0.77	0.03

ASV_76	*Desulfocapsaceae*	Unclassified	0.51	0.02
ASV_86	*Desulfocapsaceae*	*Desulforhopalus*	0.42	0.05
ASV_24	*Desulfosarcinaceae*	Unclassified	0.29	0.00
ASV_332	*Desulfosarcinaceae*	*Sva0081_sediment_group*	0.01	0.17
ASV_690	*Desulfosarcinaceae*	Unclassified	0.00	0.07
ASV_101	*DEV007*	Unclassified	0.48	0.01
ASV_1150	*DEV007*	Unclassified	0.00	0.05
ASV_131	*DEV007*	Unclassified	0.26	0.02
ASV_473	*DEV007*	Unclassified	0.07	0.01
ASV_100	*Flavobacteriaceae*	*Spongiimicrobium*	0.00	0.70
ASV_12	*Flavobacteriaceae*	*Ulvibacter*	1.66	0.08
ASV_1358	*Flavobacteriaceae*	*Pseudofulvibacter*	0.05	0.00
ASV_185	*Flavobacteriaceae*	*Arcticiflavibacter*	0.16	0.00
ASV_206	*Flavobacteriaceae*	*Actibacter*	0.14	0.00
ASV_21	*Flavobacteriaceae*	Unclassified	0.12	1.60
ASV_3	*Flavobacteriaceae*	Unclassified	2.18	0.22
ASV_309	*Flavobacteriaceae*	*Psychroserpens*	0.18	0.02
ASV_316	*Flavobacteriaceae*	*Actibacter*	0.13	0.00
ASV_435	*Flavobacteriaceae*	*Aquibacter*	0.01	0.14
ASV_47	*Flavobacteriaceae*	*Winogradskyella*	1.01	0.05
ASV_52	*Flavobacteriaceae*	*Winogradskyella*	0.65	0.03
ASV_54	*Flavobacteriaceae*	*Actibacter*	0.58	0.02
ASV_57	*Flavobacteriaceae*	*Eudoraea*	0.64	0.00
ASV_6	*Flavobacteriaceae*	Unclassified	1.36	0.05
ASV_801	*Flavobacteriaceae*	Unclassified	0.00	0.07
ASV_94	*Flavobacteriaceae*	Unclassified	0.00	0.72
ASV_98	*Flavobacteriaceae*	Unclassified	0.43	0.01
ASV_713	*Geminicoccaceae*	Unclassified	0.00	0.08
ASV_1459	*Gemmatimonadaceae*	Unclassified	0.00	0.04
ASV_280	*Geopsychrobacteraceae*	*Geopsychrobacter*	0.01	0.23

ASV_769	*Geopsychrobacteraceae*	*Desulfuromusa*	0.00	0.08
ASV_1102	*Gimesiaceae*	Unclassified	0.00	0.06
ASV_345	*Gimesiaceae*	Unclassified	0.00	0.19
ASV_431	*Gimesiaceae*	Unclassified	0.01	0.14
ASV_838	*Gimesiaceae*	Unclassified	0.00	0.07
ASV_124	*Halieaceae*	*Halioglobus*	0.40	0.03
ASV_128	*Halieaceae*	*Halioglobus*	0.36	0.03
ASV_152	*Halieaceae*	*Halioglobus*	0.37	0.03
ASV_798	*Halieaceae*	*OM60(NOR5)_clade*	0.04	0.00
ASV_187	*Hyphomicrobiaceae*	*Filomicrobium*	0.04	0.31
ASV_382	*Hyphomicrobiaceae*	*Filomicrobium*	0.00	0.17
ASV_553	*Ilumatobacteraceae*	*Ilumatobacter*	0.00	0.11
ASV_209	*Kangiellaceae*	Unclassified	0.01	0.32
ASV_1076	*Kiloniellaceae*	*Pelagibius*	0.00	0.06
ASV_665	*Kiloniellaceae*	*Pelagibius*	0.00	0.09
ASV_262	*Kiritimatiellaceae*	*R76-B128*	0.15	0.01
ASV_576	*Lentimicrobiaceae*	Unclassified	0.07	0.00
ASV_577	*Lentisphaeraceae*	*Lentisphaera*	0.08	0.01
ASV_80	*Methyloligellaceae*	*Methyloceanibacter*	0.02	0.79
ASV_934	*Methyloligellaceae*	Unclassified	0.00	0.06
ASV_358	*Microtrichaceae*	Unclassified	0.00	0.18
ASV_625	*Microtrichaceae*	*Sva0996_marine_group*	0.00	0.09
ASV_779	*Microtrichaceae*	Unclassified	0.03	0.00
ASV_1019	Unclassified	Unclassified	0.00	0.06
ASV_1032	Unclassified	Unclassified	0.00	0.06
ASV_1039	Unclassified	Unclassified	0.05	0.00
ASV_1124	Unclassified	Unclassified	0.00	0.05
ASV_1192	Unclassified	Unclassified	0.00	0.05
ASV_1229	Unclassified	Unclassified	0.00	0.05
ASV_1230	Unclassified	Unclassified	0.00	0.05

ASV_1235	Unclassified	Unclassified	0.03	0.00
ASV_1256	Unclassified	Unclassified	0.00	0.05
ASV_1298	Unclassified	Unclassified	0.00	0.05
ASV_1384	Unclassified	Unclassified	0.00	0.04
ASV_1387	Unclassified	Unclassified	0.05	0.00
ASV_1457	Unclassified	Unclassified	0.00	0.04
ASV_179	Unclassified	Unclassified	0.10	0.01
ASV_1834	Unclassified	Unclassified	0.03	0.00
ASV_200	Unclassified	Unclassified	0.03	0.29
ASV_2007	Unclassified	Unclassified	0.03	0.00
ASV_204	Unclassified	Unclassified	0.01	0.33
ASV_215	Unclassified	Unclassified	0.20	0.00
ASV_217	Unclassified	Unclassified	0.00	0.30
ASV_22	Unclassified	Unclassified	1.01	0.00
ASV_271	Unclassified	Unclassified	0.18	0.00
ASV_288	Unclassified	Unclassified	0.00	0.22
ASV_299	Unclassified	Unclassified	0.00	0.22
ASV_312	Unclassified	Unclassified	0.01	0.17
ASV_344	Unclassified	Unclassified	0.02	0.18
ASV_374	Unclassified	Unclassified	0.00	0.18
ASV_390	Unclassified	Unclassified	0.00	0.17
ASV_484	Unclassified	Unclassified	0.01	0.12
ASV_487	Unclassified	Unclassified	0.01	0.12
ASV_492	Unclassified	Unclassified	0.01	0.12
ASV_517	Unclassified	Unclassified	0.05	0.00
ASV_594	Unclassified	Unclassified	0.12	0.00
ASV_688	Unclassified	Unclassified	0.00	0.08
ASV_718	Unclassified	Unclassified	0.00	0.09
ASV_738	Unclassified	Unclassified	0.00	0.08
ASV_795	Unclassified	Unclassified	0.06	0.00

ASV_867	Unclassified	Unclassified	0.00	0.06
ASV_919	Unclassified	Unclassified	0.07	0.00
ASV_1361	*Nitrosococcaceae*	Unclassified	0.05	0.00
ASV_189	*Nitrosococcaceae*	*FS142-36B-02*	0.37	0.03
ASV_825	*Nitrosococcaceae*	*AqS1*	0.00	0.08
ASV_415	*Nitrosomonadaceae*	*IS-44*	0.01	0.15
ASV_28	*Nitrosopumilaceae*	Unclassified	0.04	1.57
ASV_398	*Nitrosopumilaceae*	Candidatus_Nitrosopumilus	0.01	0.15
ASV_507	*Nitrosopumilaceae*	Candidatus_Nitrosopumilus	0.00	0.12
ASV_575	*Nitrosopumilaceae*	Unclassified	0.00	0.11
ASV_707	*Nitrosopumilaceae*	Candidatus_Nitrosopumilus	0.00	0.09
ASV_233	*Nitrospinaceae*	Unclassified	0.00	0.29
ASV_1204	*Nitrospiraceae*	*Nitrospira*	0.00	0.05
ASV_205	*Nitrospiraceae*	*Nitrospira*	0.02	0.32
ASV_58	*Nitrospiraceae*	*Nitrospira*	0.10	0.97
ASV_981	*Nitrospiraceae*	*Nitrospira*	0.00	0.06
ASV_1151	*Opitutaceae*	*Diplosphaera*	0.00	0.05
ASV_605	*Opitutaceae*	*Diplosphaera*	0.07	0.00
ASV_438	*PHOS-HE36*	Unclassified	0.00	0.14
ASV_1103	*Phycisphaeraceae*	*SM1A02*	0.05	0.00
ASV_1505	*Phycisphaeraceae*	*SM1A02*	0.02	0.00
ASV_793	*Phycisphaeraceae*	*Urania-1B-19_marine_sediment_group*	0.06	0.00
ASV_1184	*Pirellulaceae*	*Blastopirellula*	0.04	0.00
ASV_125	*Pirellulaceae*	*Blastopirellula*	0.28	0.03
ASV_1526	*Pirellulaceae*	*Pir4_lineage*	0.00	0.03
ASV_1626	*Pirellulaceae*	*Pir4_lineage*	0.00	0.03
ASV_172	*Pirellulaceae*	*Blastopirellula*	0.36	0.03
ASV_183	*Pirellulaceae*	Unclassified	0.24	0.01
ASV_218	*Pirellulaceae*	*Bythopirellula*	0.03	0.25
ASV_224	*Pirellulaceae*	*Blastopirellula*	0.02	0.27

ASV_229	*Pirellulaceae*	*Rhodopirellula*	0.00	0.29
ASV_261	*Pirellulaceae*	*Blastopirellula*	0.02	0.20
ASV_274	*Pirellulaceae*	*Rhodopirellula*	0.14	0.01
ASV_311	*Pirellulaceae*	*Pir4_lineage*	0.13	0.00
ASV_417	*Pirellulaceae*	*Pir4_lineage*	0.01	0.14
ASV_489	*Pirellulaceae*	*Blastopirellula*	0.01	0.10
ASV_514	*Pirellulaceae*	Unclassified	0.01	0.11
ASV_556	*Pirellulaceae*	*Blastopirellula*	0.00	0.10
ASV_756	*Pirellulaceae*	*Pir4_lineage*	0.00	0.08
ASV_82	*Pirellulaceae*	*Pir4_lineage*	0.01	0.79
ASV_906	*Pirellulaceae*	Unclassified	0.00	0.05
ASV_97	*Pirellulaceae*	*Blastopirellula*	0.45	0.00
ASV_998	*Pirellulaceae*	*Blastopirellula*	0.00	0.06
ASV_337	PS1_clade	Unclassified	0.01	0.19
ASV_77	*PS1_clade*	Unclassified	0.09	0.72
ASV_103	*Rhodobacteraceae*	*Roseobacter_clade_UnclassifiedC11-7_lineage*	0.06	0.57
ASV_112	*Rhodobacteraceae*	Unclassified	0.35	0.02
ASV_160	*Rhodobacteraceae*	*Sulfitobacter*	0.04	0.37
ASV_170	*Rhodobacteraceae*	*Roseobacter_clade_UnclassifiedC11-7_lineage*	0.01	0.42
ASV_173	*Rhodobacteraceae*	Unclassified	0.02	0.40
ASV_237	*Rhodobacteraceae*	Unclassified	0.00	0.28
ASV_389	*Rhodobacteraceae*	*Limibaculum*	0.00	0.15
ASV_49	*Rhodobacteraceae*	Unclassified	0.37	0.06
ASV_643	*Rhodobacteraceae*	*Limibaculum*	0.00	0.09
ASV_66	*Rhodobacteraceae*	*Roseobacter*	0.07	0.80
ASV_675	*Rhodobacteraceae*	Unclassified	0.00	0.08
ASV_16	*Rubritaleaceae*	*Haloferula*	1.15	0.13
ASV_192	*Rubritaleaceae*	*Luteolibacter*	0.16	0.00

ASV_196	*Rubritaleaceae*	*Luteolibacter*	0.09	0.00
ASV_263	*Rubritaleaceae*	*Haloferula*	0.09	0.00
ASV_29	*Rubritaleaceae*	*Luteolibacter*	0.48	0.03
ASV_326	*Rubritaleaceae*	*Persicirhabdus*	0.00	0.20
ASV_340	*Rubritaleaceae*	*Persicirhabdus*	0.01	0.19
ASV_530	*Rubritaleaceae*	*Roseibacillus*	0.05	0.00
ASV_68	*Rubritaleaceae*	*Rubritalea*	0.37	0.02
ASV_208	*Sphingomonadaceae*	*Parasphingopyxis*	0.02	0.29
ASV_289	*Spirochaetaceae*	*Spirochaeta_2*	0.12	0.00
ASV_817	*Spongiibacteraceae*	Unclassified	0.04	0.00
ASV_849	*Thermoanaerobaculaceae*	*Subgroup_23*	0.06	0.00
ASV_275	*Thiohalorhabdaceae*	Unclassified	0.00	0.25
ASV_486	*Thiohalorhabdaceae*	Unclassified	0.00	0.13
ASV_276	*Thiomicrospiraceae*	endosymbionts	0.08	0.00
ASV_228	*Thiotrichaceae*	Unclassified	0.16	0.01
ASV_114	Unclassified *Gammaproteobacteria*	Unclassified	0.00	0.58
ASV_139	Unclassified *Gammaproteobacteria*	Unclassified	0.03	0.47
ASV_178	Unclassified *Gammaproteobacteria*	Unclassified	0.05	0.36
ASV_191	Unclassified *Gammaproteobacteria*	Unclassified	0.38	0.02
ASV_212	Unclassified *Gammaproteobacteria*	Unclassified	0.19	0.00
ASV_220	Unclassified *Gammaproteobacteria*	Unclassified	0.00	0.30
ASV_234	Unclassified *Gammaproteobacteria*	Unclassified	0.00	0.29
ASV_239	Unclassified *Gammaproteobacteria*	*Marinicella*	0.12	0.01
ASV_246	Unclassified *Gammaproteobacteria*	Unclassified	0.00	0.28

ASV_318	Unclassified *Gammaproteobacteria*	Unclassified	0.13	0.00
ASV_4	Unclassified *Gammaproteobacteria*	Unclassified	3.73	0.07
ASV_46	Unclassified *Gammaproteobacteria*	Unclassified	0.60	0.03
ASV_497	Unclassified *Gammaproteobacteria*	Unclassified	0.00	0.13
ASV_512	Unclassified *Gammaproteobacteria*	*Marinicella*	0.09	0.00
ASV_543	Unclassified *Gammaproteobacteria*	Unclassified	0.01	0.11
ASV_623	Unclassified *Gammaproteobacteria*	Unclassified	0.06	0.00
ASV_865	Unclassified *Gammaproteobacteria*	*Marinicella*	0.05	0.00
ASV_950	Unclassified *Gammaproteobacteria*	Unclassified	0.00	0.06
ASV_971	Unclassified *Gammaproteobacteria*	Unclassified	0.00	0.06
ASV_118	*Woeseiaceae*	*Woeseia*	0.39	0.01
ASV_133	*Woeseiaceae*	*Woeseia*	0.02	0.53
ASV_180	*Woeseiaceae*	*Woeseia*	0.00	0.40
ASV_257	*Woeseiaceae*	*Woeseia*	0.00	0.26
ASV_279	*Woeseiaceae*	*Woeseia*	0.00	0.24
ASV_298	*Woeseiaceae*	*Woeseia*	0.01	0.21
ASV_324	*Woeseiaceae*	*Woeseia*	0.02	0.18
ASV_343	*Woeseiaceae*	*Woeseia*	0.00	0.19
ASV_386	*Woeseiaceae*	*Woeseia*	0.01	0.15
ASV_503	*Woeseiaceae*	*Woeseia*	0.00	0.12
ASV_618	*Woeseiaceae*	*Woeseia*	0.00	0.10
ASV_63	*Woeseiaceae*	*Woeseia*	0.03	0.97
